# Genome Wide Associations of Growth, Phenology, and Plasticity Traits in Willow [*Salix viminalis* (L.)]

**DOI:** 10.3389/fpls.2019.00753

**Published:** 2019-06-12

**Authors:** Henrik R. Hallingbäck, Sofia Berlin, Nils-Erik Nordh, Martin Weih, Ann-Christin Rönnberg-Wästljung

**Affiliations:** ^1^Department of Plant Biology, Uppsala BioCenter, Linnean Centre for Plant Biology, Swedish University of Agricultural Sciences, Uppsala, Sweden; ^2^Department of Crop Production Ecology, Linnean Centre for Plant Biology, Swedish University of Agricultural Sciences, Uppsala, Sweden

**Keywords:** SRC willows, biomass, phenology, plasticity, association mapping, *Salix viminalis* L., GWAS, marker-assisted selection

## Abstract

The short rotation biomass crop willow (*Salix* genera) has been of interest for bioenergy but recently also for biofuel production. For a faster development of new varieties molecular markers could be used as selection tool in an early stage of the breeding cycle. To identify markers associated with growth traits, genome-wide association mapping was conducted using a population of 291 *Salix viminalis* accessions collected across Europe and Russia and a large set of genotyping-by-sequencing markers. The accessions were vegetatively propagated and planted in replicated field experiments, one in Southern Sweden and one in Central Sweden. Phenology data, including bud burst and leaf senescence, as well as different growth traits were collected and measured repeatedly between 2010 and 2017 at both field environments. A value of the plasticity for each accession was calculated for all traits that were measured the same year in both environments as the normalized accession value in one environment subtracted by the corresponding value in the other environment. Broad-sense accession heritabilities and narrow-sense chip heritabilities ranged from 0.68 to 0.95 and 0.45 to 0.99, respectively for phenology traits and from 0.56 to 0.85 and 0.24 to 0.97 for growth traits indicating a considerable genetic component for most traits. Population structure and kinship between accessions were taken into account in the association analyses. In total, 39 marker-trait associations were found where four were specifically connected to plasticity and interestingly one particular marker was associated to several different plasticity growth traits. Otherwise association consistency was poor, possibly due to accession by environment interactions which were demonstrated by the low structure adjusted accession correlations across environments (ranging from 0.40 to 0.58). However, one marker association with biomass fresh weight was repeatedly observed in the same environment over two harvest years. For some traits where several associations were found, the markers jointly explained over 20% of the accession variation. The result from this study using a population of unrelated accessions has given useful information about marker-trait associations especially highlighting marker-plasticity associations and genotype-by-environment interactions as important factors to take account of in future strategies of *Salix* breeding.

## Introduction

Fast growing trees for bioenergy has been of interest since the 1980s and lately the increasing demand of non-fossil fuels has further put fast growing woody biomass production into focus. Fast growing shrubby *Salix* species and more recently also *Populus* species have been used in a short rotation coppice (SRC) system with harvests every 3–5 years from the same plants in order to continuously have a high biomass production (Weih, 2004). Breeding programs for *Salix* and *Populus* has been developed both in Europe and North America to fulfill the goals of high producing and well adapted plant material for cultivation (Karp and Shield, 2008; Kuzovkina et al., 2008; Karp et al., 2011; Stanton et al., 2010; Serapiglia et al., 2013). In Europe *Salix viminalis, S. dasyclados, and S. schwerinii* have been the main willow species used for SRC plantations (Karp et al., 2011; Hanley and Karp, 2014). *Salix viminalis* has a history of cultivation to provide raw material for basketry and for stabilization of river banks across Europe and has been domesticated to some degree due to trading of clonal material between countries resulting in successive expansion of the species geographic distribution (Lascoux et al., 1996; Kuzovkina et al., 2008; Berlin et al., 2014). Despite this the *Salix* species of interest for SRC are still at a low level of domestication implying substantial genetic variation within species and a high degree of individual heterozygosity (Berlin et al., 2011, 2014). As a result, recurrent selection programs have developed cultivars with increased biomass production compared to old varieties originating from wild collections (Åhman and Larsson, 1994; Larsson, 1998; Kuzovkina et al., 2008; Karp et al., 2011).

An important aspect of breeding for improved local adaptation is how environmental differences influence the performance of the different genotypes, also called genotype-by-environment interaction (G × E). The response of a genotype across environments reflect the plasticity of the genotype and its possibility to survive and grow in different environmental conditions (Bradshaw, 1965; Schlichting, 1986; Wu, 1998). Studying and identifying genetic factors that regulate plasticity of traits is highly interesting from an evolutionary and ecological point of view since it ultimately sets the limit for the distribution of a species (Stapley et al., 2010; Tétard-Jones et al., 2011; Savolainen et al., 2013). For plant breeding it is also crucial to understand how plant material can be transferred and used in different environmental conditions in order to set up breeding zones within which material can be transferred without losing in productivity (Via and Lande, 1985; Nicotra et al., 2010). To identify the genetic basis of plasticity *per se* would make it possible to breed for more or less plastic individuals. Many studies have been conducted to reveal the genetic base and the genetic regulation of plasticity in plants (see reviews by Nicotra et al., 2010; El Soda et al., 2014). In *Populus* quantitative trait loci (QTL) mapping have gained more insight into the genetic background of plasticity (Rae et al., 2008; Fabbrini et al., 2012) and in Rae et al. (2008) evidence for heritable genetic variation for plasticity traits was shown and these traits were in many cases regulated by QTL different from QTL regulating the traits themselves. Also, in *Salix* species, studies show clonal differences in plasticity across environments and identified QTL for plasticity demonstrates a genetic basis for the plasticity traits (Berlin et al., 2014, 2017).

To further increase the genetic gain in a recurrent selection breeding program, selection tools using molecular markers could be included in the breeding process (Allwright and Taylors, 2016). A first step toward marker-assisted selection (MAS) has been to dissect the genetic basis for different important breeding traits and identify markers influencing the trait variation. This has been accomplished with several molecular marker techniques and various statistical approaches giving different genomic resolution. For instance, QTL mapping can be performed, where regions in the genome important for the trait variation are indicated, whereas in association mapping either variation in trait candidate genes or genome wide single nucleotide polymorphism (SNP) markers are used to attempt the identification of the actual gene that regulate the trait variation. A drawback with QTL-studies is that the variation seen in the mapping population originates from a limited set of parents and thus restricts the generality of the results. In association mapping studies, on the other hand, where preferably unrelated individuals are sampled from natural populations of the species, any detected genotype-phenotype association would have a more general applicability for the species since more alleles are studied (Khan and Korban, 2012). Because recombination at an evolutionary timescale has made the extent of linkage disequilibrium very short, many thousands of markers are needed for a genome wide association study (GWAS) to be able to find an association (Neale and Kremer, 2011).

With the aim of identifying markers for MAS, different QTL studies have been conducted in *Populus* and *Salix* species to understand the genetics of different disease resistance traits (Jorge et al., 2005; Rönnberg-Wästljung et al., 2008; Hanley et al., 2011; Samils et al., 2011), phenology and biomass related traits (Dillen et al., 2009; Rae et al., 2009; Wullschleger et al., 2011; Berlin et al., 2014, 2017; Ghelardini et al., 2014). More recently association mapping studies have been performed that identified putative causative genes for growth and phenology traits in *Salix* and in *Populus* species (Allwright and Taylors, 2016; Fahrenkrog et al., 2016; Hallingbäck et al., 2016; Carlson et al., 2019) and for traits related to chemical wood properties (Wegrzyn et al., 2010; Guerra et al., 2013; Porth et al., 2013; Muchero et al., 2015; Carlson et al., 2019), all that are of potential interest for breeding purposes.

*Salix viminalis* is an excellent model species for trees due to the possibility of propagating genotypes vegetatively and thus simplifying the establishment and analysis of common garden experiments across environments. *Salix viminalis* also has a very short generation time of one or two years which facilitates genetic studies and breeding. Furthermore, *S. viminalis* is dioecious (like other species of the Salicaceae family) and thus 100% outcrossing giving a high genetic diversity and heterozygosity (Berlin et al., 2014). Linkage disequilibrium for *S. viminalis* has been estimated to vary from 0.5 to 0.6 with a decrease to 0.2 at around 4000 bp, a slower decrease than in *S. schwerinii* which could be of importance for GWAS and the possibility to identify associations (Berlin et al., 2011). With a population of *S. viminalis* consisting of 291 accessions earlier genetically and phenotypically described (Berlin et al., 2014) and studied for associations using a candidate gene approach (Hallingbäck et al., 2016), we here perform a GWAS with markers positioned across the whole genome and biomass traits. The main objective of this study was to identify genotype-phenotype associations for different biomass related traits as well as for plasticity traits. We report on multi-year and multi-environment association analysis on phenology and growth traits as well as plasticity across environments.

## Materials and Methods

### Plant Material and Field Experiments

The association mapping population includes *S. viminalis* accessions that originates from the United Kingdom, Sweden, Belgium, Germany, and Western Russia (Berlin et al., 2014), and from natural willow stands in the Czechia (Trybush et al., 2012). The different accessions were collected at latitudes between 48.1°N to 62.4°N and longitudes from 4.8°W to 104.3°E. In spring 2009, field experiments were established at two different environments in Sweden; Pustnäs, south of Uppsala (59.80°N, 17.67°E, 25 m ASL) and in Svalöv, southern Sweden (55.56°N, 13.06°E, 75 m ASL). The two environments differ with respect to temperature during spring (April) and autumn (September, October) months (Table 1) where the town Lund close to Svalöv, showed consistently higher mean month temperatures (difference ranging from 0.9 to 3.6°C) than that of Uppsala close to Pustnäs. Also, the soil in the Pustnäs experiment is a sandy soil with approx. 2% clay while the soil in the Svalöv experiment is a loam with 15–25% of clay content.

**Table 1:** Monthly mean temperatures (°C) during the growing seasons of the years in which assessments were made in the Salix field experiments in Pustnäs (near Uppsala) and Svalöv (near Lund).

		Apr	May	Jun	Jul	Aug	Sep	Oct
2010	Uppsala	5.7	11.4	15.2	20.7	16.6	11.2	5.3
	Lund	7.4	10.5	15.2	20.9	17.6	13.1	7.8
2011	Uppsala	9.0	11.8	17.2	18.8	16.9	13.4	7.6
	Lund	10.3	12.3	16.7	17.6	16.8	14.6	9.4
2012	Uppsala	4.5	11.8	13.4	17.2	16.1	11.8	5.8
	Lund	6.6	13.2	14.3	17.8	17.9	13.9	8.5
2013	Uppsala	4.5	14.0	16.3	18.1	17.2	12.2	7.4
	Lund	6.5	13.7	15.8	18.8	17.7	13.1	10.8
2014	Uppsala	7.0	10.9	13.7	20.5	16.9	12.5	8.5
	Lund	9.1	12.8	15.8	20.8	17.0	15.1	11.7
2015	Uppsala	7.0	9.8	14.1	16.6	17.5	12.8	6.2
	Lund	7.9	10.7	14.2	17.3	18.6	13.8	9.5
2016	Uppsala	5.8	12.7	–^a^	18.1	16.2	14.5	6.6
	Lund	7.3	14.7	17.4	18.1	17.3	16.6	8.7
2017	Uppsala	4.5	11.0	15.1	17.1	16.2	12.6	7.1
	Lund	6.8	13.1	16.2	16.8	17.4	13.8	10.7
**Average**	**Uppsala**	**6.0**	**11.7**	**15.0**	**18.4**	**16.7**	**12.6**	**6.8**
**2010–2017**	**Lund**	**7.7**	**12.6**	**15.7**	**18.5**	**17.5**	**14.3**	**9.6**


*Source: Swedish Meteorological and Hydrological Institute, www.smhi.se. ^*a*^Data missing.*

A total of 388 accessions were planted with 20 cm long cuttings at a density of 15,000 ha^-1^ at both field experiments. Each accession was represented by six clonal replicates per experiment arranged in a randomized complete block design. The spacing was 130 cm between rows and 50 cm between plants within rows. To avoid border effects, two rows of the accession 78183 were planted outside the experimental plants. Later genetic analyses revealed that several of the accessions were the same clone (Berlin et al., 2014; Hallingbäck et al., 2016) and subsequently they were collapsed and treated as one accession leading to a final number of 321 accessions. Except for the first year when no fertilization was made to the field experiments, fertilization corresponding to 70 kg N ha^-1^ was applied each year. The plants were cut back in winter 2009/2010, 2013/2014, 2014/2015, and 2016/2017 at both experiments and an additional cut back was made during winter 2010/2011 at Svalöv. Further details about the association mapping collection are found in Berlin et al. (2014).

### Phenotypic Measurements

Leaf bud burst was assessed on each individual plant in Pustnäs during spring 2010, 2011, 2013, and 2014 using a scale between 1 and 5 where 1 equals no sign of bud growth and 5 equals the most developed bud burst stage, with one or more leaves growing perpendicular to the shoot axis (Weih, 2009). The bud burst assessments were repeated during a period between late April and middle of May in order to find the time point for each year where the most variation in bud burst was observed (May 5, 2010; April 15, 2011; May 2, 2013; April 29, 2014, respectively). These timepoints were chosen as traits for further analyses (BB10, BB11, BB13, BB14; Table 2). Leaf senescence and abscission were visually assessed in Pustnäs on November 4 and 5, 2010 (LS10), on October 31, 2011 (LS11), and in Svalöv on November 9, 2016 (LS16) according to a leaf senescence index (LSI) from 0 to 4 with 0 = no leaves left on the plant (100% abscission) and 4 = more than 80% green leaves (∼10% abscission) (Ghelardini et al., 2014). During winter 2010/2011 and 2015/2016 two growth traits closely related to total biomass (Nordh and Verwijst, 2004) were assessed at Pustnäs, while at Svalöv these measurements were taken only during winter 2015/2016. The number of shoots (Nsh11, Nsh16) was counted and the diameters on all shoots above 105 cm from the ground were measured. Mean diameter (MeanD11, MeanD16) and the summed basal area (SumBA11, SumBA16) were calculated and used for analysis. During cut back of all plants winter 2013/2014 and 2016/2017 fresh weight (FW14, FW17) and total number of shoots (Nsh14, Nsh17) of each plant were taken at both experiments.

**Table 2:** Summary statistics for the traits; abbreviations, measurement units, number of plants measured, overall arithmetic means (per plant), and individual standard deviations (SD) for Pustnäs and Svalöv.

			Pustnäs				Svalöv			
Trait	Abbr.	Unit	Age of shoot/root	No. obs.	Mean	SD	Age of shoot/root	No. obs.	Mean	SD
**Spring phenology traits**					
Budburst 2010	BB10	Score	0/1	2238	3.28	0.57	–	–	–	–
Budburst 2011	BB11	Score	1/2	2251	2.37	0.72	–	–	–	–
Budburst 2013	BB13	Score	3/4	1502	2.83	0.70	–	–	–	–
Budburst 2014	BB14	Score	0/5	1856	3.45	0.70	–	–	–	–
**Autumn phenology traits**					
Leaf senescence 2010	LS10	Score	1/2	2255	2.29	0.76	–	–	–	–
Leaf senescence 2011	LS11	Score	2/3	2247	1.56	0.71	–	–	–	–
Leaf senescence 2016	LS16	Score	–	–	–	–	2/8	2055	1.52	0.66
**Biomass growth traits**					
No. of shoots 2011	Nsh11	No.	1/2	2258	9.59	5.23	–	–	–	–
No. of shoots 2014	Nsh14	No.	4/5	2258	6.02	3.47	3/5	2097	6.38	4.09
No. of shoots 2016	Nsh16	No.	1/7	2156	18.26	10.71	1/7	2002	11.66	7.51
No. of shoots 2017	Nsh17	No.	2/8	2241	34.39	14.73	2/8	2068	17.29	9.19
Mean shoot diameter 2011	MeanD11	mm	1/2	2245	7.33	1.58	–	–	–	–
Mean shoot diameter 2016	MeanD16	mm	1/7	2153	4.56	1.06	1/7	2002	5.27	1.33
Summed shoot basal area 2011	SumBA11	mm^2^	1/2	2245	842.10	547.24	–	–	–	–
Summed shoot basal area 2016	SumBA16	mm^2^	1/7	2153	384.28	313.09	1/7	2002	330.80	300.70
Fresh weight 2014	FW14	kg	4/5	2255	4.96	3.50	3/5	2097	1.46	1.20
Fresh weight 2017	FW17	kg	2/8	2243	2.34	1.72	2/8	2068	2.09	1.67


*Data from year 2010 to 2013 at Pustnäs have been presented earlier (Hallingbäck et al., 2016) but are included for comparison.*

### Genotyping-by-Sequencing

From unique accessions of the study population, young leaves were sampled (approximately 200 mg) and DNA was extracted following a CTAB-protocol described in Pucholt et al. (2017), which in turn was a modification of the protocol from Brunner et al. (2001). DNA-extracts were genotyped with the genotyping-by-sequencing method (GBS) at the Genomic Diversity Facility, Cornell University, Ithaca, NY, United States. In a manner much similar to that of Elshire et al. (2011), the DNA was digested by the *Ape*KI restriction endonuclease, ligated to sample specific barcode adapter sequences and subsequently sequenced on an Illumina Next-generation sequencing (NGS) platform (Illumina Inc., San Diego, CA, United States). Sequence reads and polymorphisms were provided at the Genomic Diversity Facility by running the Tassel GBS analysis pipeline v. 3.0.166 (Glaubitz et al., 2014) using the available genome sequence of the close relative *Salix purpurea* as a mapping reference (Zhou et al., 2018; *Salix purpurea* v1.0, DOE-JGI^1^). For the 291 accessions for which sampling, DNA extractions, and genotyping were successful, DNA sequence sites showing polymorphisms were identified. The resulting genotype data were merged by Tassel ver. 4.3.7, provided to us as Variant Call Format files (VCF) and is stored at a publicly available repository (Hallingbäck et al., 2019). The polymorphisms consisted mainly of biallelic SNPs (1,235,800) plus smaller numbers of alleged triallelic SNPs (231,512), biallelic indels (67,435), and triallelic indel/SNP combinations (21,047).

For each putative polymorphic site, diploid genotypes were called by finding the maximum likelihood for the observed distribution of haplotype sequence reads (Hohenlohe et al., 2010). But in order to avoid biases and to ensure sufficient calling accuracy, genotypes were called only provided a read depth of at least five at any particular site and accession. Otherwise the genotype was set as *missing*. Assuming an Illumina genotyping error rate of 0.1% this procedure implies a diploid genotype calling accuracy above 97%. The polymorphism data produced by GBS was thereafter merged with genotype data from 1290 SNPs previously developed for this population (Hallingbäck et al., 2016). Mapping of the old SNPs to the *S. purpurea* genome was performed by Blast (*e* < 10^-9^) whereupon all polymorphic sites were sorted according to increasing chromosome number and position with respect to the reference. The polymorphic sites were then filtered based on data completeness and on minor allele frequency (MAF) depending on intended downstream use. All the above-mentioned processing steps were performed with VCFtools ver.0.1.12b. Furthermore, to reduce the number of sites produced by erroneous merging of paralog or repetitive sequences, all sites for which the frequency of heterozygotes exceeded 70% were removed using a custom perl script. Assuming Hardy–Weinberg equilibrium, even triallelic sites should never exhibit heterozygosity frequencies above 66.7%.

### Structure and Kinship Analysis

In order to take into account the effects of population structure and relatedness, structure and relatedness was inferred. This was done using a dataset which used all polymorphic sites with >95% called genotypes and a MAF of >1% resulting in total 19,243 markers. Using this dataset, a kinship matrix (*K*) for the sample population was estimated in accordance with Loiselle et al. (1995). Moreover, population structure was investigated by applying an admixture model with correlated allele frequencies within populations using the software STRUCTURE ver. 2.3.4 (Pritchard et al., 2000; Falush et al., 2003). In order to avoid potentially adverse effects of linkage disequilibrium between markers the dataset used for this was reduced by selecting every fifth marker (3,848 markers) out of those used for kinship estimation. The number of clusters assumed (*k*) was set to vary from one to ten. The length of the burn-in period was set to 20,000 iterations and the subsequent sample recording period was set an additional 40,000 iterations. For each *k*, ten replicates were run. Similarly to previous studies using smaller marker sets (Berlin et al., 2014), the optimal value of *k* was found to be 4 by examination of the maximum log-likelihood at convergence and on Evanno’s Δ*k* statistics (Evanno et al., 2005). A graphical illustration of the ancestry of individual accessions to the population structure clusters is shown in Supplementary Figure S1.

### Development of Accession Estimators

To obtain unbiased estimators (BLUEs) for each accession and trait, phenotypic data was subjected to variance decomposition applying linear mixed models (LMM) with the statistics software ASReml 3.0 (Gilmour et al., 2009). All analyses were made on untransformed data since, even though the distributions for some of the number of shoots, summed shoot basal area and fresh weight deviated from normal, the results from the analysis with transformed data did not differ from analyses with original data. The mixed linear model applied was:

$$y_{ijkl}=\mu +b_{i}+c_{j}+{\left(r\times p\right)}_{kl}+e_{ijkl}\;\;\;\;\;\;\;\;\;(1)$$

where *y_ijkl_* is the phenotypic trait value in the *i*th block for the *j*th accession located at row *k* and position *l*. The overall mean is denoted as μ while *b* signifies the fixed block effect, *c* the fixed accession effect, *r* ×*p* the random interaction between rows and positions (spatial term) and *e* the random residual. Random effects were assumed to be independent except for the spatial term (*r* ×*p*) which was restricted to follow a two-dimensional first order autoregressive correlation structure (Cullis and Gleeson, 1991) across plant rows and positions. Thus accession estimators (*y_es_*) adjusted for block and spatial environmental effects were obtained from the effect estimates as *y_es,j_ = 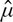 + ĉ_j_* and were used in subsequent analyses.

In addition to the estimators *per se*, the (broad sense) accession estimator heritability ($H_{\text{c}}^{2}$)was also estimated for each trait and for that purpose *c* was instead considered to be random. $H_{\text{c}}^{2}$ may serve as an indicator of accession estimator precision and was calculated from the estimated accession variance ($\sigma_{\text{c}}^{2}$) and error variance ($\sigma_{\text{e}}^{2}$) components as:

$$H_{\text{c}}^{2}=\frac{\sigma_{\text{c}}^{2}}{\sigma_{\text{c}}^{2}+\frac{1}{n}\sigma_{\text{e}}^{2}}\;\;\;\;\;\;\;\;\;(2)$$

where *n* is the harmonic mean of the number of measured plants per accession.

For each clone, plasticity variables of traits were calculated as the standardized (mean = 0, standard deviation = 1) BLUE values in Pustnäs subtracted with standardized BLUE values in Svalöv when traits were measured the same year (Nsh14, Nsh16, Nsh17, MeanD16, SumBA16, FW14, FW17).

### Association Mapping Marker Dataset

Polymorphic sites used as markers for the association mapping itself were required to be biallelic, have clear genotypic calls for at least 75% of the individuals and MAF > 5%. Furthermore, any adjacently located indel polymorphisms that would cancel each other out (erroneously called in the GBS pipeline) were removed. The LD (*r^2^*) between closely situated markers (according to the *S. purpurea* reference) was estimated using a maximum likelihood method (Hill, 1974) implemented in the function “LD” in the *Genetics* library of R. This calculation was performed using a *sliding window* scheme where the LD was estimated between each marker and its 100 closest neighbor markers – upstream and downstream. In this way, certain groups of markers were found to be redundant (*r^2^* > 0.99) and only one representative marker, having the highest genotype call percentage in that group, were retained for further analysis. Redundant markers were nonetheless kept in a separate *accessory marker* list to enable future reference. In all, the fully filtered association mapping dataset comprised 19,411 polymorphic markers. Missing marker genotypes were imputed using the LD-kNNi method as implemented by Money et al. (2015) in the software *LinkImpute*. The number of nearest relatives (*k*) and the number of SNP markers within a 10 Mb distance that were in closest LD with the marker to be imputed (*l*), were optimized by *LinkImpute* and were determined as *k* = 15 and *l* = 5.

### Association Mapping Analysis and Model Selection

Using the imputed marker dataset and the previously calculated accession estimators (*y_es_*), a second series of multilocus linear mixed model (MLMM) analyses were performed, again using ASReml 3.0 (Gilmour et al., 2009). The number of accessions having both the required genotype data and accession BLUE estimators were 291 and 288 for Pustnäs and Svalöv, respectively (288 accessions in common). The MLMM were applied in separate analyses for each trait at the Pustnäs experiment, the Svalöv experiment and for the plasticity traits as:

$$y_{\text{es}}=Fq+Sg+Zu+\;e_{\text{es}}$$

where **y_es_** is the vector form of the BLUE accession estimators (*y_es,j_*), **q** and **g** are the vectors of fixed population ancestry and SNP genotype effects, respectively, **u** is the vector of random *chip additive genetic effects* (Speed et al., 2012; Kruijer et al., 2015) linked to the marker-based kinship matrix **K**, and **e_es_** is the vector of the random residual effects. The design matrix **S** constitutes the individual genotypes for one or several analyzed SNPs as separate and independent factors, implying genetic effects of the form: **g** = [*g_AA,_*_1_ *g_Aa,_*_1_ *g_aa,_*_1__…_ *g_AA,n_ g_Aa,n_ g_aa,n_*]*^T^* for markers 1 to *n* included in the model, each featuring the genotypes *AA, Aa*, and *aa*, respectively. **F** and **Z** link the respective individual ancestry proportion and additive chip effect to its observation. All effects were considered to be statistically independent except for the chip additive genetic effects whose variance was assumed to be structured as $Var\left(u\right)=2\sigma_{A}^{2}K$ where $\sigma_{\text{A}}^{2}$ is the chip additive genetic variance. The number of markers included in the **Sg** model factor was determined by consecutive scans of all markers using the forward–backward stepwise model selection approach of Segura et al. (2012). Structure component effects **(q)** and chip additive genetic variance ($\sigma_{\text{A}}^{2}$) were re-estimated for each analysis. In the forward model selection phase, the SNP with the largest -*log*(*p*) values (Wald-*F* test) was selected and during the backward phase, the included SNP with the smallest *-log*(*p*) value was eliminated. Forward selections of up to five markers were made and the model with the maximum number of markers all exhibiting Bonferroni corrected *p-*values of 0.2 or less (*p <* 1.03 × 10^-5^) was reported and further analyzed. The percentage variance explained of any marker *i* added to the model ($R_{\delta i}^{2}$), was calculated as the difference between the percentage variance explained of all fixed effects already in the model including marker *i* ($R_{incl}^{2}$) and the corresponding percentage of variance explained of a reduced model including the same fixed effects except for marker *i* ($R_{excl}^{2}$). Please note that $R_{incl}^{2}$ and $R_{excl}^{2}$ comprise fixed effects of ancestral structure **(q)** as well as all marker genotype effects **(g)** of stronger statistical significance than marker *i* in order to discount *R*^2^ inflation due to collinearity (e.g., due to LD). Both $R_{incl}^{2}$ and $R_{excl}^{2}$ were estimated using the fixed effect estimators for their respective models as advocated by Nakagawa and Schielzeth (2013). Finally LD correlations were estimated between all significantly trait-associated markers using the “LD” function of the *Genetics* R-package.

For each identified marker association, all genes in the region 3,000 bp up- and downstream the marker position were checked in the *S. purpurea* genome (Zhou et al., 2018; *Salix purpurea* v1.0, DOE-JGI, see footnote 1). The size of the region to check genes were based on LD estimates of *S. viminalis* (Berlin et al., 2011), which show a decay of LD to a non-significant value of 0.2 at a distance of 4,000 bp from the marker.

### Chip Genetic Parameter Estimation

For the association analyses the full model previously presented was used, but in order to estimate other genetic parameters adjusted for ancestral structure such as the chip narrow sense heritability ($h_{\text{s}}^{2}$) and accession correlations between traits or environments (*r_s_*), analyses were made were the model component of individual markers **(Sg)** was excluded.

$$y_{\text{es}}=Fq+Zu+\;e_{\text{es}}$$

Thus, for each trait, $h_{\text{s}}^{2}$ was calculated from the estimated chip additive genetic and residual variance components ($\sigma_{e,es}^{2}$) as:

$$h_{s}^{2}\;=\;\frac{\sigma_{A}^{2}}{\sigma_{A}^{2}+\sigma_{e,es}^{2}}$$

Furthermore, in order to structure adjusted estimate accession correlations (*r_s_*) between traits thereby also assessing the potential occurrence of genotype-by-environment (G × E) interactions, multivariate extensions of the previously described model were made treating each trait-year-environment combination as a separate variate (e.g., Burdon, 1977). Accession correlations were based on the summed variances of the random effect model terms for any trait 1 or 2 (e.g., $\sigma_{A1}^{2}+\sigma_{e,es1}^{2}$) and the correspondingly summed covariances between trait 1 and 2 (*σ_A12_ + σ_e,es12_*) as:

$$r_{s}=\frac{\sigma_{A12}+\sigma_{e,es12}}{\sqrt{(\sigma_{A1}^{2}+\sigma_{e,es1}^{2})\left(\sigma_{A2}^{2}+\sigma_{e,es2}^{2}\right)}}$$

Estimation errors for all genetic parameters were calculated using the delta method based on the Taylor series approximation (Casella and Berger, 2002).

## Results

### Trait Means and Variation

**FIGURE 1:**
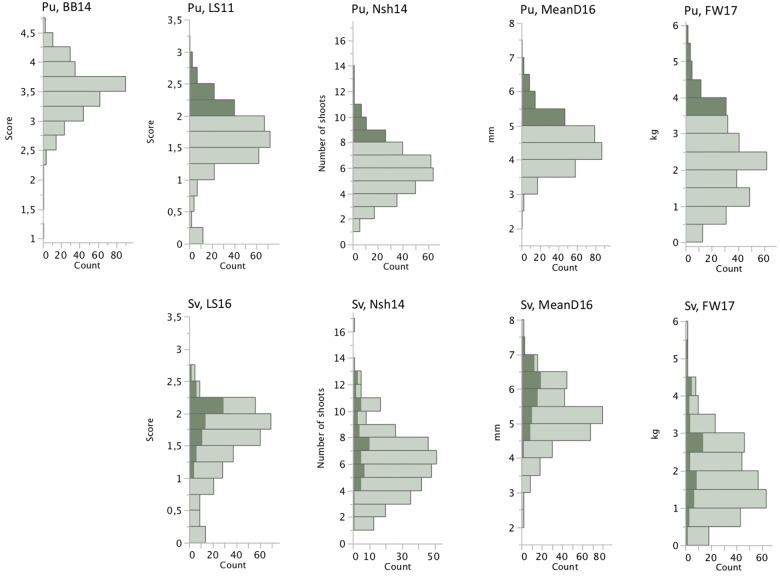
Phenotypic distributions of a subset of the measured traits based on arithmetic means of each accession in the two field experiments, Pustnäs and Svalöv. Shaded bars indicate the accessions with highest trait mean values in Pustnäs and the values for the corresponding trait and the same accessions in Svalöv.

The association mapping population showed large variation for all measured traits both at Pustnäs and at Svalöv (Table 2 and Figure 1). The number of shoots and fresh weight during 2014 were measured and harvested when the experiments had different shoot ages, 4 and 3 years for the Pustnäs and Svalöv experiments, respectively. This was reflected by the higher mean fresh weight in Pustnäs compared to Svalöv (Table 2). During 2016 there were some tendencies for a higher number of shoots with lower mean diameter at the Pustnäs experiment compared to Svalöv, which also resulted in a higher summed basal area in Pustnäs. Still, these values were accompanied by large standard deviations and might not reflect a true difference between the experiments. For 2017 a comparison between the experiments show larger number of shoots in Pustnäs but during this year the difference in fresh weight between experiments was small.

### Marker Variation

**FIGURE 2:**
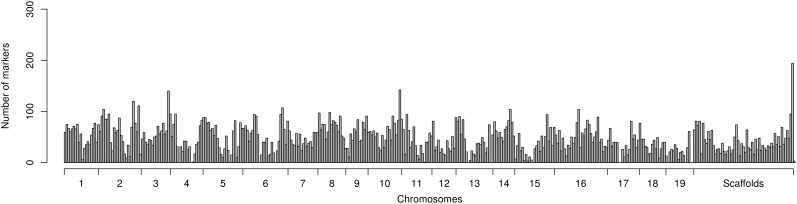
Distribution of markers mapped across the genome sequence of *Salix purpurea* genome available to the public. Each marker distribution bin encompasses 1 Mb of the genome sequence.

The marker dataset used for association mapping analyses consisted of 19,411 polymorphic markers fairly evenly distributed across the genome (Figure 2). Of these markers 18,273 (94%) were genotyped using the genotyping-by-sequencing approach while the remainder 1,138 markers were available from previous candidate gene exploration (Hallingbäck et al., 2016). Mapping to a chromosome of the established *S. purpurea* genome was possible for 16,943 markers (87%) while the remainder 2,468 markers could only be mapped to 569 incompletely integrated sequence in smaller sized scaffolds (Figure 2). 17,853 (92%) of the markers were regular biallelic SNPs while the remainder 1,558 (8%) were single nucleotide indels.

### Genetic Parameters

Table 3 shows broad-sense accession estimator heritabilities ($H_{\text{c}}^{2}$) as well as structure-adjusted narrow sense chip heritabilities ($h_{\text{s}}^{2}$) for each trait in both Pustnäs and Svalöv. In general, $H_{\text{c}}^{2}$-estimates were medium ($H_{\text{c}}^{2}$ between 0.56 and 0.69) to high ($H_{\text{c}}^{2}$ above 0.70) reflecting a considerable genetic component underlying the accession variation. Autumn phenology traits showed especially high $H_{\text{c}}^{2}$-estimates ranging from 0.89 to 0.95. Also, chip heritabilities in Svalöv were high ($h_{\text{s}}^{2}$ above 0.73) independent of the trait while in Pustnäs estimates varied considerably (from 0.24 for SumBA11 to 0.97 for MeanD16) for growth traits. The phenology $h_{\text{s}}^{2}$-estimates in Pustnäs were high (0.75–0.99) with BB10 being the only exception ($h_{\text{s}}^{2}$ at 0.45). As the relationship between accessions was generally low, estimation errors of the chip heritabilities are considerable.

**Table 3:** Broad sense accession estimator heritability ($H_{\text{c}}^{2}$) and structure-adjusted narrow-sense chip heritabilities ($h_{\text{s}}^{2}$) estimated for each trait measured at Pustnäs and Svalöv along with the structure-adjusted accession correlations (r_s_) between these two field experiments.

Trait	Pustnäs		Svalöv		
		
	$H_{\text{c}}^{2}$	$h_{\text{s}}^{2}$	$H_{\text{c}}^{2}$	$h_{\text{s}}^{2}$	*r_s_*
**Spring phenology traits**					
BB10	0.68 (0.03)	0.45 (0.19)	–	–	–
BB11	0.87 (0.01)	0.99^∗^	–	–	–
BB13	0.82 (0.02)	0.88 (0.13)	–	–	–
BB14	0.69 (0.03)	0.75 (0.15)	–	–	–
**Autumn phenology traits**					
LS10	0.95 (0.01)	0.99^∗^	–	–	–
LS11	0.91 (0.01)	0.82 (0.12)	–	–	–
LS16	–	–	0.89 (0.01)	0.94 (0.10)	–
**Biomass growth traits**					
Nsh11	0.59 (0.04)	0.40 (0.17)	–	–	–
Nsh14	0.62 (0.03)	0.52 (0.17)	0.60 (0.03)	0.73 (0.15)	0.50 (0.05)
Nsh16	0.74 (0.02)	0.54 (0.17)	0.70 (0.03)	0.74 (0.15)	0.40 (0.05)
Nsh17	0.80 (0.02)	0.44 (0.17)	0.69 (0.03)	0.77 (0.15)	0.57 (0.04)
MeanD11	0.71 (0.03)	0.31 (0.15)	–	–	–
MeanD16	0.85 (0.01)	0.97 (0.10)	0.78 (0.02)	0.88 (0.12)	0.58 (0.04)
SumBA11	0.56 (0.04)	0.24 (0.18)	–	–	–
SumBA16	0.72 (0.02)	0.72 (0.15)	0.70 (0.03)	0.75 (0.14)	0.39 (0.05)
FW14	0.74 (0.02)	0.48 (0.18)	0.63 (0.03)	0.75 (0.13)	0.43 (0.05)
FW17	0.77 (0.02)	0.64 (0.16)	0.68 (0.03)	0.81 (0.12)	0.43 (0.05)


*Estimation errors are given in parentheses. ^∗^Estimation error unavailable as the estimate is based on fixed variance components at REML convergence.*

**FIGURE 3:**
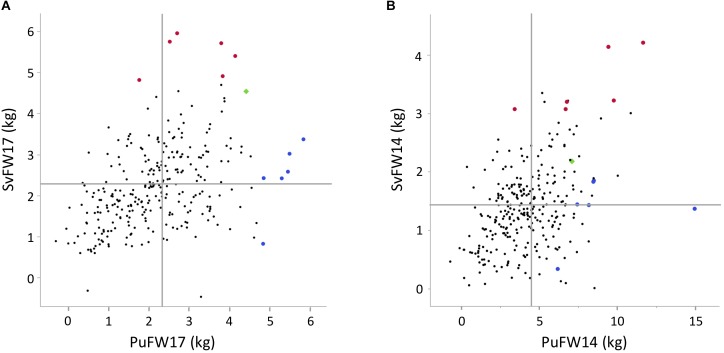
Plots of BLUE accession estimators for different traits between the two field experiments, **(A)** FW17, **(B)** FW14. Red enlarged dots represent the best accessions for FW17 in Svalöv, the blue enlarged dots in Pustnäs, and the green square represent an accession that has high FW17 at both experiments. Horizontal lines indicate the BLUE means in Svalöv and vertical line indicate BLUE means in Pustnäs.

Structure adjusted accession correlation estimates across environments were low-to-moderate for the different traits (0.39–0.58) indicating G × E-interactions (Table 3). This was also reflected in Figure 1 where the accessions with highest mean values in Pustnäs (shaded) do not always have the highest values in Svalöv. Plots of accession BLUEs in Figure 3 illustrate for fresh weight (FW) 2017 and 2014 the limited structure-adjusted accession correlations between experiments where the accessions with highest FW in Pustnäs were not the accessions with highest FW in Svalöv and vice versa. Still some accessions showed a stability in FW17 across experiments (Figure 3A).

For structure adjusted accession correlations between traits at the same environment but measured during different years, estimates were moderate-to-high (0.45–0.94) for the number of shoots (Nsh), estimated biomass (SumBA) and fresh weight (FW) both at Pustnäs and Svalöv (Table 4). For FW this is also illustrated in Figure 3 where, especially in Svalöv, the accessions with the highest FW in 2017 also in many cases exhibited the highest FW in 2014. Bud burst in Pustnäs did not show high correlations between measurements (0.15–0.52). In particular the 2013 bud burst assessment correlated poorly with the assessment made in 2010 as well as in 2014. Bud burst in 2010 and 2014 was scored from the stump close to ground level which implies an environment different from the budburst scoring in 2013 which was performed on 3-year-old stems on a height level of one or several meters above the ground. This difference would be the most reasonable explanation for the low *r_s_*-estimates. The structure-adjusted accession correlations between different growth traits were above 0.50 in 50% of the possible correlations in Pustnäs and in 82% of the correlations in Svalöv while correlation magnitudes between the two different phenology traits measured in Pustnäs never exceeded 0.5 (-0.07 to 0.15) (Table 4). Very few accession correlations between phenology and growth traits at any of the environments were above 0.5 (2% in Pustnäs and none in Svalöv).

**Table 4:** Accession correlations (r_s_) adjusted for population structure between traits measured in Pustnäs (above diagonal) and Svalöv (below diagonal).

Trait	BB10	BB11	BB13	BB14	LS10	LS11	LS16	Nsh11	Nsh14	Nsh16	Nsh17	MeanD11	MeanD16	SumBA11	SumBA16	FW14	FW17
BB10	x	**0.41**	**0.23**	**0.52**	0.00	0.04	–	**0.33**	**0.33**	**0.26**	**0.26**	0.11	**0.15**	**0.33**	**0.28**	**0.25**	**0.29**
BB11	–	x	**0.50**	**0.41**	-0.07	-0.00	–	0.06	0.07	**0.26**	**0.22**	**0.33**	**0.28**	**0.31**	**0.34**	**0.36**	**0.38**
BB13	–	–	x	**0.15**	0.03	0.10	–	0.02	0.01	0.08	0.08	0.09	0.07	0.11	0.13	**0.18**	**0.16**
BB14	–	–	–	x	**0.15**	**0.14**	–	**0.33**	**0.39**	**0.40**	**0.45**	**0.30**	**0.33**	**0.50**	**0.48**	**0.46**	**0.50**
LS10	–	–	–	–	x	**0.59**	–	0.03	0.04	0.06	0.07	**0.19**	**0.14**	**0.20**	0.12	**0.13**	0.11
LS11	–	–	–	–	–	x	–	**0.13**	**0.13**	**0.13**	**0.19**	**0.28**	**0.28**	**0.32**	**0.22**	**0.31**	**0.23**
LS16	–	–	–	–	–	–	x	–	–	–	–	–	–	–	–	–	–
Nsh11	–	–	–	–	–	–	–	x	**0.91**	**0.45**	**0.64**	–**0.12**	–0.00	**0.60**	**0.30**	**0.31**	**0.31**
Nsh14	–	–	–	–	–	–	**0.20**	–	x	**0.45**	**0.67**	–0.07	0.00	**0.63**	**0.30**	**0.36**	**0.32**
Nsh16	–	–	–	–	–	–	**0.16**	–	**0.78**	x	**0.81**	**0.21**	**0.32**	**0.53**	**0.86**	**0.49**	**0.83**
Nsh17	–	–	–	–	–	–	**0.18**	–	**0.81**	**0.89**	x	**0.20**	**0.18**	**0.62**	**0.65**	**0.61**	**0.72**
MeanD11	–	–	–	–	–	–	–	–	–	–	–	x	**0.65**	**0.57**	**0.47**	**0.58**	**0.49**
MeanD16	–	–	–	–	–	–	**0.33**	–	**0.30**	**0.37**	**0.16**	–	x	**0.50**	**0.68**	**0.61**	**0.62**
SumBA11	–	–	–	–	–	–	–	–	–	–	–	–	–	x	**0.65**	**0.75**	**0.64**
SumBA16	–	–	–	–	–	–	**0.28**	–	**0.68**	**0.88**	**0.71**	–	**0.63**	–	x	**0.69**	**0.93**
FW14	–	–	–	–	–	–	**0.28**	–	**0.77**	**0.80**	**0.71**	–	**0.54**	–	**0.87**	x	**0.76**
FW17	–	–	–	–	–	–	**0.35**	–	**0.69**	**0.84**	**0.75**	–	**0.57**	–	**0.94**	**0.89**	x


*Estimated magnitude larger than twice the size of their standard errors are given in bold.*

### Association Mapping

In total, across all traits and environments as well as for the plasticity of traits, 39 marker-trait associations of significance (*p* < 0.2 after Bonferroni correction) were found (Tables 5, 6 and Supplementary Figures S2–S8). Of these associations five were based on recessive effects linked to a homozygote group with only one accession while an additional six associations were based on recessive effects linked to a homozygote group of five accessions with very close kinship (𝜃 ranging from 0.620 to 0.672) that originated from beyond the Ural Mountains. These particular associations should thus be treated with skepticism. However, discounting such associations ten associations between markers and phenology traits were nonetheless found in Pustnäs (Table 5). For growth traits we also found eight such associations in Pustnäs. In Svalöv no significant association was found for the only phenology trait (LS16), but for growth traits six marker-trait associations not affected by the aforementioned issues, were identified.

**Table 5:** Marker-trait associations satisfying the chosen significance threshold (*p* < 1.03 × 10^-5^) observed from analysis of the Pustnäs or Svalöv experiments plus marker type, alleles (common/rare), and rare allele frequencies for the corresponding markers.

Marker	Chr	Pos	Type	Alleles	Freq	*p*	*R*^2^ (%)
**Pustnäs**					

**BB10**					
S1_332378951^a^	18	4285907	SNP	T/C	0.084	7.88 × 10^-8^	8.4
S1_20013804	16	20013804	SNP	A/T	0.323	1.13 × 10^-6^	5.8
S1_196933747	9	6102489	SNP	G/C	0.131	9.66 × 10^-6^	5.2
Total							19.4
**BB11**							0.0
**BB13**					
S1_223421640	11	823582	SNP	T/A	0.156	1.60 × 10^-6^	6.1
S1_122829941	5	13435194	SNP	T/C	0.296	2.12 × 10^-6^	5.2
S1_398080262	1232^c^	22975	SNP	A/G	0.079	9.72 × 10^-7^	5.2
S1_55653105	2	5911772	SNP	A/G	0.074	2.28 × 10^-6^	4.3
Total							20.8
**BB14**					
S1_107621523	4	16888940	SNP	A/C	0.218	1.18 × 10^-7^	9.2
S1_136537234^a^	6	4445445	Indel	T/–	0.064	1.91 × 10^-8^	5.8
S1_226299259	11	3701201	SNP	A/G	0.175	1.43 × 10^-6^	5.8
S1_238507049	11	15908991	SNP	C/T	0.227	2.83 × 10^-6^	5.7
Total							26.6
**LS10**					
S1_263152945^b^	13	9456239	SNP	C/T	0.137	5.35 × 10^-9^	9.2
S1_183614214	8	8686605	Indel	C/–	0.086	8.17 × 10^-6^	1.8
Total							11.0
**LS11**					
S1_263152945^b^	13	9456239	SNP	C/T	0.137	7.63 × 10^-8^	9.6
**Nsh11**					
S1_39473243^a^	1	9304403	SNP	T/C	0.179	1.70 × 10^-12^	8.2
S1_26031342	16	26031342	SNP	T/A	0.438	2.32 × 10^-6^	5.6
S1_356219816	19	12917686	SNP	T/A	0.387	2.88 × 10^-6^	4.9
S1_364538411	215^c^	262103	SNP	A/G	0.127	5.00 × 10^-6^	5.0
S1_84875818	3	10814216	SNP	A/G	0.204	9.33 × 10^-6^	5.8
Total							29.6
**Nsh14**							0.0
**Nsh16**					
S1_51767602	2	2026269	SNP	T/C	0.253	2.29 × 10^-6^	6.0
S1_5924474	16	5924474	SNP	A/T	0.266	6.37 × 10^-6^	6.5
Total							12.5
**Nsh17**							0.0
**MeanD11**					
S1_133339294^b^	6	1247505	SNP	G/A	0.065	2.70 × 10^-14^	15.2
S1_409355463	1560^c^	46981	SNP	A/G	0.143	1.86 × 10^-6^	6.1
Total							21.3
**MeanD16**					
S1_203858009^b^	10	340916	SNP	A/G	0.069	3.98 × 10^-8^	18.1
S1_280431011	14	5689249	SNP	C/T	0.285	2.30 × 10^-7^	6.0
S1_121751392	5	12356645	Indel	T/–	0.179	8.42 × 10^-7^	2.3
Total							26.4
**SumBA11**							0.0
**SumBA16**					
S1_203903296^b^	10	386203	SNP	T/A	0.072	9.58 × 10^-7^	12.9
**FW14**		
S1_15451811^b^	16	15451811	SNP	A/C	0.086	5.12 × 10^-7^	8.7
**FW17**							0.0
**Svalöv**							
**LS16**							0.0
**Nsh14**							
S1_328299990^a^	18	206946	SNP	T/G	0.065	5.72 × 10^-6^	3.9
S1_89445373	3	15383771	SNP	G/T	0.062	5.66 × 10^-6^	3.8
S1_146130713	6	14038924	SNP	C/T	0.058	4.97 × 10^-6^	4.9
S1_399409820	1265^c^	56257	Indel	–/T	0.129	5.57 × 10^-6^	5.8
Total							18.4
**Nsh16**							0.0
**Nsh17**							0.0
**MeanD16**							0.0
**SumBA16**							0.0
**FW14**							
S1_361520786	117^c^	271270	SNP	T/C	0.058	1.44 × 10^-6^	6.1
S1_31799294	1	1630454	SNP	T/C	0.052	7.26 × 10^-6^	5.9
Total							12.0
**FW17**							
S1_361520786	117^c^	271270	SNP	T/C	0.058	9.65 × 10^-6^	5.6


*^*a*^The association is dependent on the effect of a single BB-homozygote individual and should be treated with caution. ^*b*^The association dependent on a very close cluster of BB-homozygotes originating beyond the Ural mountains and should be treated with caution. ^*c*^The underlying marker is located in the indicated scaffold not yet successfully incorporated into the *Salix purpurea* genome assembly.*

**Table 6:** Marker-trait associations satisfying the chosen significance threshold (*p* < 1.03 × 10^-5^) observed for calculated plasticity of traits between the Pustnäs and Svalöv experiments plus marker type, alleles (common/rare), and rare allele frequencies for the corresponding markers.

Marker	Chr	Pos	Type	Allele	Freq	*p*	*R*^2^ (%)
**Nsh14**					
S1_246135575	12	6178441	Indel	–/C	0.258	1.05 × 10^-6^	8.5
**Nsh16**		
S1_201052459^a^	9	10221201	SNP	T/C	0.065	1.17 × 10^-6^	8.8
**Nsh17**							0.0
**MeanD16**							0.0
**SumBA16**		
S1_246135575	12	6178441	Indel	–/C	0.258	8.67 × 10^-6^	7.4
**FW14**							0.0
**FW17**		
S1_246135575	12	6178441	Indel	–/C	0.258	1.53 × 10^-6^	8.6


*^*a*^The association is dependent on the effect of a single BB-homozygote individual and should be treated with caution.*

**FIGURE 4:**
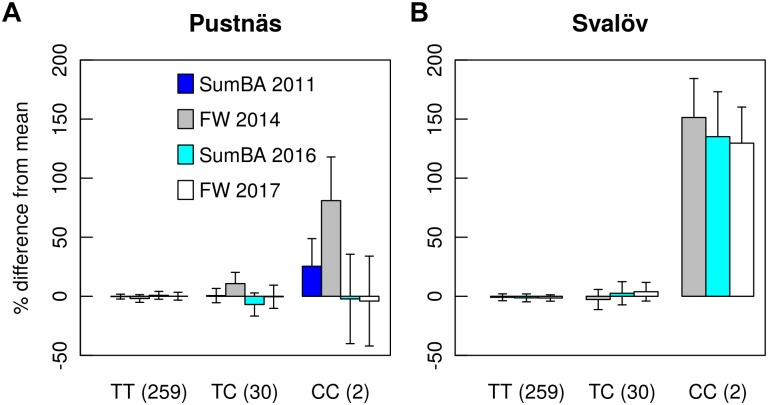
Genotype effects for the marker S1_361520786 on summed basal area of shoots (SumBA) and fresh weight (FW) at Pustnäs **(A)** and Svalöv **(B)** as percentages related to the population mean. The values in parentheses after each genotype group describe the number of accessions belonging to that group.

When considered individually, trait-associated markers explained from 1.8 to 18.1% of the accession variation but when discounting for association effects dependent on single homozygotes or to the five accessions beyond the Ural Mountains, any single trait-marker association explained up to 9.2% of the accession variation. Taking into account the variation explained by all marker associations for a trait, $R_{tot}^{2}$-estimates could reach 20.8% (BB13, BB14) when many markers were identified. Significant markers were rarely associated to several traits simultaneously but the marker S1_361520786 was a notable exception being connected to fresh weight in Svalöv during both harvests in 2014 and in 2017 (Table 5). With respect to this marker the rare allele homozygotes exhibited 130 to 151% better growth in terms of biomass traits (SumBA, FW) in Svalöv (Figure 4B) in comparison to the population mean. Indeed this marker showed effects of similar tendencies for SumBA11 and FW14 also in Pustnäs (25–80%; Figure 4A) although this trend was not statistically significant (*p >* 0.2 after Bonferroni correction). The interpretation of the marker S1_361520786 effects also requires some caution because only two rare allele homozygotic accessions constituted the basis for the apparent recessive effect raising the possibility that the effects may be overestimated. Nonetheless the effect cannot be dismissed as a case of cryptic structure because the two homozygotic accessions involved were almost completely unrelated according to kinship estimates (𝜃 = 0.010).

**FIGURE 5:**
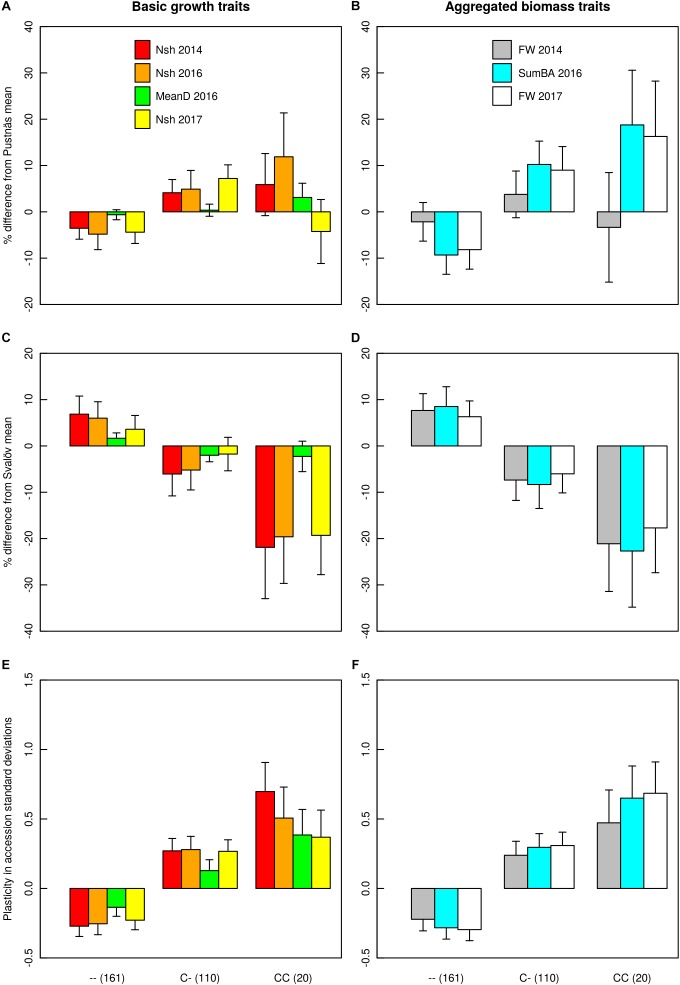
Genotype effects for the marker S1_246135575 on number of shoots (Nsh), mean shoot diameter (MeanD), summed basal area of shoots (SumBA), and fresh weight (FW) at Pustnäs **(A,B)**, Svalöv **(C,D)** and for the corresponding differences (plasticity) between Pustnäs and Svalöv **(E,F)**. Genotype effects at Pustnäs and Svalöv are described as percentages relative to the population mean, while plasticity effects are given in unit standard deviations of BLUE accession estimators. The values in parentheses after each genotype (class) describe the number of accessions having that genotype (class).

A total of four associations were found for plasticity traits. Interestingly, one specific marker on chromosome 12 (S1_246135575) was associated both with the plasticity of number of shoots 2014, SumBA16 and FW17 explaining from 7.4 to 8.6% of the accession variation (Table 6 and Supplementary Figures S7, S8). In Figure 5 the effects of this indel marker is shown for different genotypes both with respect to the plasticity traits (subplots E,F) as well as for the biomass traits themselves at the two field experimental locations (subplots A–D). The addition of an insert-C allele appeared to confer a substantial negative effect in Svalöv for SumBA16 and FW17 (-12 to -17%, Figure 5D) while exerting a positive effect on the corresponding traits in Pustnäs (7–20%, Figure 5B). A similar although less obvious pattern was also observed for the number of shoots (Figure 5A,C). In terms of plasticity this implied an overall positive additive effect of an added insert-C allele ranging from 0.10 to 0.61 accession standard deviations (Figure 5E,F).

In order to know whether the multiple marker-trait associations detected in this study could reflect a smaller set of unique associations, we estimated linkage disequilibrium (LD) correlations between all markers that had significant trait associations. However, such correlation estimates were generally very low indicating no or little LD (Supplementary Figure S9). Only 14 marker pairs (out of 595) showed LD correlations with magnitudes above 0.2. Seven of those cases, including the case with the strongest LD correlation (*r* = 0.35), were between markers whose trait association effects depended on the rare homozygotic accession group originating beyond the Ural mountain range and these were viewed with skepticism anyway. In summary, there was no substantive evidence that the other associations constituted multiple reflections of a lower set of unique associations.

All genes in the region 3000 bp up- and downstream the associated markers are reported in Supplementary Table S1. Many of the genes were connected to membrane transport or to transcription factors. The marker that in Svalöv repeatedly was identified for FW14 and FW17 (S1_361520786) was situated in a gene connected to translational initiation factor 4B. Translational initiation factors are involved in a number of processes connected to growth and to interaction with different stresses and could thus be important factors in plant improvement (Dutt et al., 2015). The marker identified for plasticity of different growth traits (S1_246135575) was in a gene resulting in an integral membrane HPP family protein which is a large group of proteins that involved in many different processes connected to membranes. Both markers were in the non-coding region of the genes.

## Discussion

In this study, we investigated associations between a genome wide set of markers and growth and phenology related traits, measured repeatedly during several years and in two different environments. Genome wide association studies with biomass traits has never been conducted in *Salix viminalis* but recently within the same genera in *S. purpurea* (Carlson et al., 2019). Previously, Hallingbäck et al. (2016) used a candidate gene approach and identified associations between growth traits and phenology traits and markers in *S. viminalis*. An obvious drawback with this approach is that only genes with known functions will be analyzed. Compared with previous QTL-mapping studies, (e.g., Berlin et al., 2014, 2017; Ghelardini et al., 2014) the identified associations in the current GWAS and in the candidate gene study will have a better resolution and be closer to the causative genes due to the shorter LD in a population of unrelated individuals compared to biparental populations (Neale and Kremer, 2011; Khan and Korban, 2012). On the other hand, in a population with short LD, a GWAS requires a large number of markers to cover the complete genome to identify associations (Khan and Korban, 2012). In *S. viminalis* LD has earlier been estimated to drop to low non-significant values within 4000 bp from a marker which is longer compared to the closely related species *S. schwerinii* (Berlin et al., 2011) and show promise for possibilities to identify associations with a reasonable number of markers. Here we have identified 19,411 SNPs and single nucleotide indel markers from a GBS-effort, quite evenly distributed across the genome, to use in a first attempt of GWAS with biomass traits in *Salix viminalis*.

The population of *S. viminalis* used in the study contains accessions collected from the British Isles in the west throughout Europe into Russia as well as clonal material from breeding programs in England and in Sweden (Berlin et al., 2014). It could be regarded as a population well representing Western and Central Europe while being a bit sparse in the Eastern distribution of the species. The population could also, based on GBS marker data (this study) and microsatellite data (Berlin et al., 2014), be divided into four subpopulations where one subpopulation includes the accessions with Russian origin. One complication was that the kinship analyses revealed another substructure within this Russian population where five accessions originating from diverse places beyond the Ural Mountain range were all indicated to be in very close kinship (Hallingbäck et al., 2016 and present study). As the population size was rather limited (291) it was not considered prudent to eliminate accessions only due to this reason. Instead, throughout the remainder of the discussion such associations whose effects are solely dependent on single accessions or the accessions beyond the Ural Mountains (footnotes in Tables 5, 6) will not be seriously considered even though they may appear strongly significant or show high *R^2^*-estimates.

Substantial phenotypic variation could be seen for all traits at both environments with mostly high broad-sense accession estimator heritability ($H_{\text{c}}^{2}$) values for phenology traits and medium-to-high for growth traits. Also, the narrow-sense chip heritabilities ($h_{\text{s}}^{2}$) were considerable with values up to 0.99 for phenology while estimates were more variable for growth traits (0.24–0.97) depending of trait and environment. This general trend with higher heritability estimates for phenology traits compared with growth traits was also observed earlier (Rönnberg-Wästljung, 2001) where two large factorial crossings of *S. viminalis* with clones of Swedish and Polish origin were used. This also seems to be a general trend in many boreal and temperate tree species (e.g., McKown et al., 2014; Guerra et al., 2016). Genetic correlation estimates between phenology and biomass traits were low indicating different genetic background of the different types of traits and suggest that these traits could be selected for independently of each other. In an earlier biparental QTL study (Berlin et al., 2017), phenology QTL overlapped in some cases with QTL for different biomass traits which could indicate a common genetic background but it should nonetheless be cautioned that the broad QTL regions identified in that study contains many different genes raising the possibility that the QTL overlap merely reflected linkage.

Taking into consideration the structure of the population as well as the kinship between accessions and corrections for multiple testing we could in total identify 25 associations across environments, traits and years whose genotypic effects could not be ascribed to either a single homozygotic accession or to the tightly related group of trans-Ural accessions. For bud burst (BB13, BB14) and for the number of shoots in 2011 (Nsh11) in Pustnäs, such associated markers explained over 20% of the total accession variation. GWAS in different *Populus* species identified markers for different wood traits (Porth et al., 2013; Du et al., 2015b) that in total explained above 20% of the variance for each trait. Du et al. (2015b) also identified markers for different growth traits as stem diameter, tree height and stem volume, that together explained from 24 to 33% of the accession variation. In any case it should be noted that even 20% explained variation is still a limited proportion in comparison to the percentage of accession variation explained by genetics overall, namely $H_{\text{c}}^{2}$ which varied from 59% to 82% for BB13, BB14, and Nsh11. This implies that, given the current sample size (291 accessions), a substantial portion of the genotypic variation was still unaccounted for in terms of individual marker-trait associations. In addition, we also found three associations, not connected to single homozygote or trans-Ural accessions, connected to plasticity of biomass traits.

Most of the associations individually explained a limited portion of the total accession variation ranging from 2.3 to 9.2%. Again, considering the previously mentioned large portions of genotypic variation unexplained by marker associations, and that the limited size of the study population would allow only the marker-trait associations with the greatest *R^2^* to be detected with any confidence, the results of this study agree with the notion that biomass and phenology traits are regulated by a multitude of small effect QTL, a concept originally suggested by Fisher (1918). This is furthermore in agreement with previously reported GWAS results from *Populus* studies (McKown et al., 2014; Du et al., 2015a) and further supported by meta-analyses for a number of quantitative traits in tree species in general (e.g., Hall et al., 2016).

For traits studied in Pustnäs and Svalöv there was only one case where the marker-trait association was stable from one year to another (FW14 to FW17 in Svalöv). In line with this result, aggregated biomass traits, such as summed basal area and fresh weight, showed consistently strong accession correlations between years of measurement. For other traits, accession correlations were generally lower and associations were less stable and unique for the trait measurement. Some stable associations were found in the previous candidate gene study (Hallingbäck et al., 2016) where, for example a SNP situated in the ELF3b-gene was consistently identified across years and environments. But also in that investigation, most of the associations were unique. Similar results were found for *S. purpurea* where most of the identified associations were unique with some few consistent across years and environments (Carlson et al., 2019). The general trend also in *Populus* is that unique as well as some stable associations are found, e.g., Muchero et al. (2013, 2015) identified several associations for different wood traits that were stable across environments and between developmental stages.

It is notable that the association of SNP S1_361520786 to both FW14 and FW17 in Svalöv was largely dependent on a considerable recessive effect linked to two unrelated accessions that were homozygous for the rare C-allele of the SNP (Figure 4). Therefore this association certainly need more examination in order to be confirmed and one should remain skeptical of the large effect which may be considerably overestimated (e.g., Beavis, 1994; Xu, 2003). Nonetheless, one should not entirely disregard associations that are dependent on few individuals or on low allele frequency markers, since the effect may still become a considerable factor if frequency of the rare allele can be increased by MAS or by conventional breeding. Indeed, similar results were detected in the previous candidate gene study using the same population, where an interesting SNP in the ELF3b-gene showed highly significant effects connected to few individuals in one of the homozygote classes (Hallingbäck et al., 2016). It should be emphasized that the chief reason why the observation of this association was not repeated in this study was that one of the homozygote accessions of the SNP of ELF3b was not successfully genotyped by GBS likely leading to its non-significance in the current analyses. In summary, low frequency alleles might still play an important role to explain the variation in a complex trait and for the missing heritability but are often discarded in association analysis (Brachi et al., 2011; Fahrenkrog et al., 2016).

The two field environments exhibited differences in climate, especially in spring (April) and in autumn (September, October) temperature, and in soil type but both represent typical environments used for practical cultivation of SRC *Salix*. We found no common marker associations between environments for the different growth or phenology traits. This finding could of course be an effect of the statistical power of the analysis being insufficient to detect one and the same association repeatedly but could also be due to G × E-interactions between the Pustnäs and Svalöv. Indeed, the moderate accession correlations between Pustnäs and Svalöv indicate substantial G × E-interactions between the two environments and Hallingbäck et al. (2016) also identified G × E-interactions between Pustnäs and a contrasting site situated in the United Kingdom (Woburn). Similar patterns with few common associations between environments was also seen for bud burst using a population of *Populus balsamifera* grown in common garden experiments at two locations in Canada, while bud set shared more common associations between locations (Olson et al., 2013). These circumstances together with the non-stability of markers will have important implications for the eventual conduct of MAS where different sets of markers have to be used in different environments for breeding of adapted plant material.

Given the observed G × E-interactions in the current study, we estimated the plasticity of each genotype by examining the standardized differences in trait accession BLUEs between Pustnäs and Svalöv and then mapped this difference for associations. One specific marker (S1_246135575) was identified to associate with the plasticity of three different growth and biomass traits (Nsh14, SumBA16 and FW17) thus being remarkably consistent. This marker explained around 8% of the accession variation suggesting that we have identified an important element of the genetic base for growth trait plasticity. Furthermore, the genotypic effect of this marker showed that the rare C-insert allele produced an increase in biomass traits in one environment while a decrease in the other environment. Interestingly these effects behaved in a largely additive manner (Figure 5). The main climatic difference between the two field environments are in spring and autumn where Svalöv, the more southern environment, has higher temperatures and thus giving a longer growing season. In addition, the Svalöv environment also has a soil with more clay content which most probably also have an influence of the biomass growth. The marker identified is clearly influenced by environmental differences, an effect that could be used in breeding as indicated below.

### Breeding Implications

This is one of the first association mapping study in *Salix* using a GWAS approach with biomass traits, that successfully has identified a set of candidate markers connected to growth and phenology traits as well as to plasticity of traits. Each marker does not explain much of the total phenotypic variation but the effect of the marker may still be substantial. Adding several markers together explain a greater amount of the accession variation. In a breeding perspective these markers and preferably a set of markers with specific allele combinations could be of value and interesting to use in the selection of parents as well for selection of individuals in early stages of the breeding cycle. An early selection of seed plants would reduce the number of plants for field testing and thus also make the breeding more cost effective. Some of the marker-trait associations identified were connected to a homozygote genotypic class with few individuals of large effect, these markers need to be validated through, for example, crossings of heterozygotes and study of the phenotypes in the segregating offspring, i.e., multiple family QTL mapping (Ukrainetz et al., 2008).

Even though the accession correlation across environments for the different traits was moderate indicating G × E-interactions, we still observed specific accessions that performed well in both environments. Such accessions could be of use for breeding toward stable performance across environments with plasticity close to zero. Another alternative to handle the G × E is to divide the area of cultivation into different breeding zones utilizing the very best performing accessions in each region and thus breed material for more specific use (Namkoong et al., 1988). As an example, the marker connected to plasticity gave a considerable effect for biomass traits and number of shoots with different effect dependent of environment and could be used to select for stability (heterozygotes) or for different alleles to increase the frequency of homozygotes for better performance in selected environments.

Nonetheless, given the general impression that the studied traits are likely regulated by a multitude of loci each exerting relatively minor effects and the considerable G × E-interactions encountered in this study, it appears difficult to efficiently utilize any specific set of few markers to perform MAS (see also Grattapaglia et al., 2018). A different alternative in this regard would be to perform MAS by genotyping breeding populations using large sets of markers that would utilize the LD with a multitude of small effect QTL, so called *genomic selection* (Meuwissen et al., 2001; Harfouche et al., 2012; Desta and Ortiz, 2014). The marker-trait associations observed in this or other studies could offer candidate targets for directed genotyping efforts, e.g., by developing SNP arrays. By enlisting markers linked to previously detected QTL into such arrays, further links, based on causality or LD, could be established between markers and QTL. Thereby the selection accuracy of genomic prediction models could be increased and the efficiency of genomic selection would be improved.

The results from this study using a population of unrelated accessions have given basic information about marker-trait associations that are of importance and could be used for future strategies and breeding of *Salix*. In general, individual associations explained a limited proportion (<10%) of the accession variation although, in concert, associations with budburst and number of shoots occasionally explained about 20%. Associations were generally not repeated across measurement years and environments, due to limited statistical power or to the substantial G × E-interactions observed. Still, one particular association for fresh weight was observed to be consistent across years in the Svalöv experiment and for the plasticity of growth traits between Pustnäs and Svalöv, another association was detected which was consistent across years and traits to a greater degree. Further examination of these associations should contribute to an improved understanding of the genetic architecture of important traits in *Salix* and facilitate the development of marker-assisted breeding methods for this species.

## Author Contributions

A-CR-W, SB, and MW planned and designed the research. N-EN and A-CR-W performed the field measurements. HH and A-CR-W analyzed the data and wrote the manuscript. All authors read and approved the manuscript.

## Conflict of Interest Statement

The authors declare that the research was conducted in the absence of any commercial or financial relationships that could be construed as a potential conflict of interest.

## Footnotes

1 http://phytozome.jgi.doe.gov/pz/portal.html#%20!info?%20alias=Org_Spurpurea
