# Supplementary material for: Genome Wide Associations of Growth, Phenology, and Plasticity Traits in Willow [Salix viminalis (L.)]
Source: Front Plant Sci. 2019 Jun 12;10:753. doi: 10.3389/fpls.2019.00753 (PMC6582754; doi:10.3389/fpls.2019.00753)
Supplement: Supplementary file 1 [file Image_1.pdf]

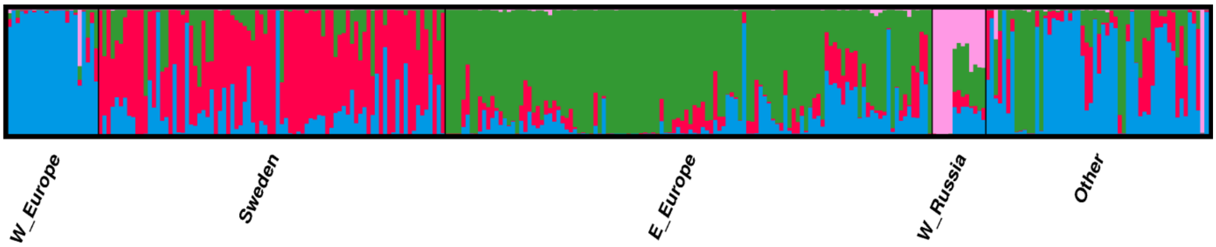

Figure S1. Illustration of the ancestry of individual accessions according to population structure clusters where the x-axis details the geographic region where the accession was sampled, “Other” implies that sampling origin was unknown (see also Berlin et al. 2014). The colours signify proportional ancestry to a specific cluster (blue for W European, red for Swedish, green for E European and pink for Russian clusters respectively).

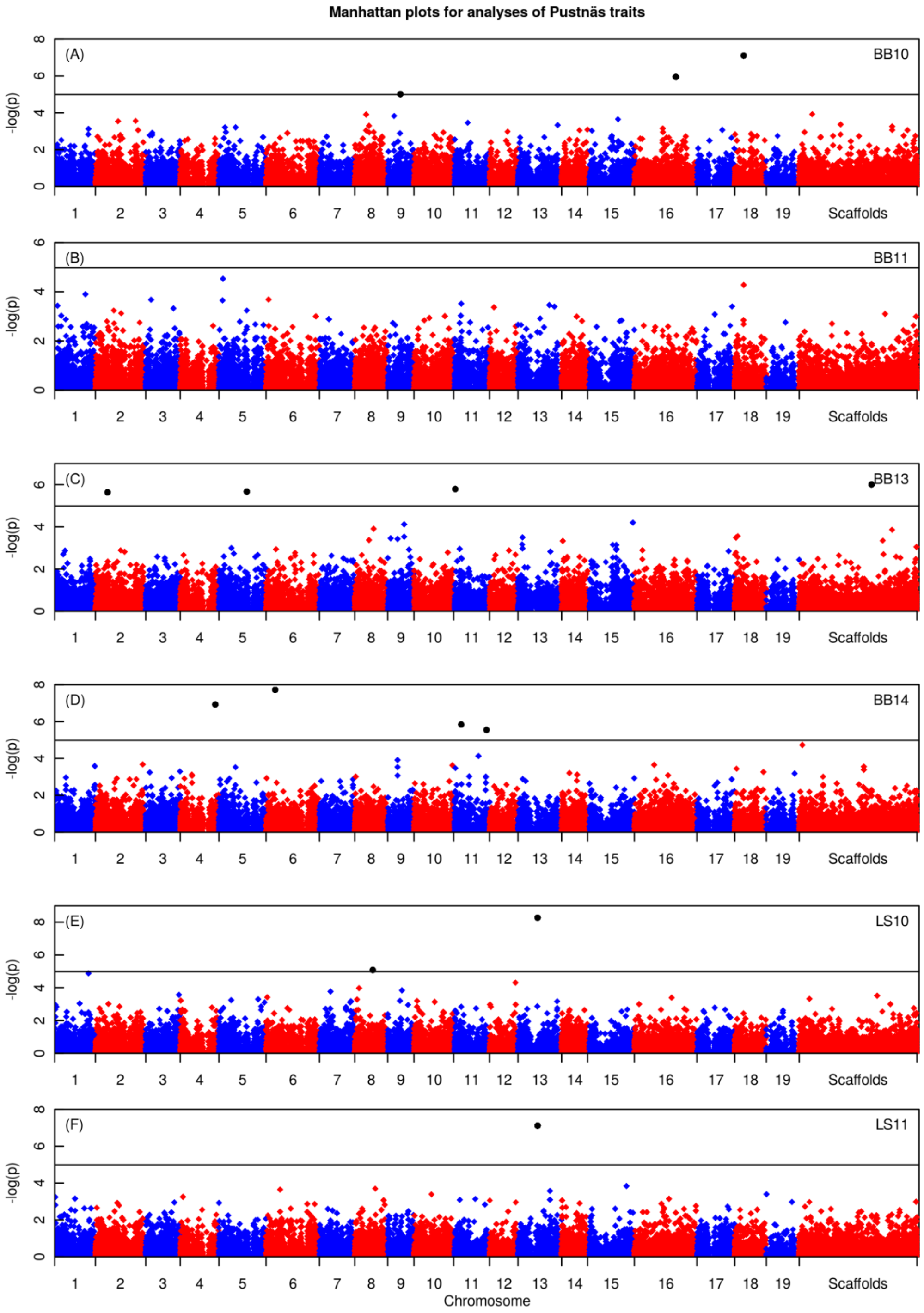

Figure S2. Manhattan plots showing the negative logarithms of *p*-values (*-log(p)*) of marker associations to budburst (BB, subplots A-D) and leaf senescence (LS, subplots E-F) during the years 2010, 2011, 2013 and 2014 in Pustnäs. The chosen significance threshold (p=1.03·10^{-5}) is shown as a black line and associations included in the final selected model are highlighted as black circles.

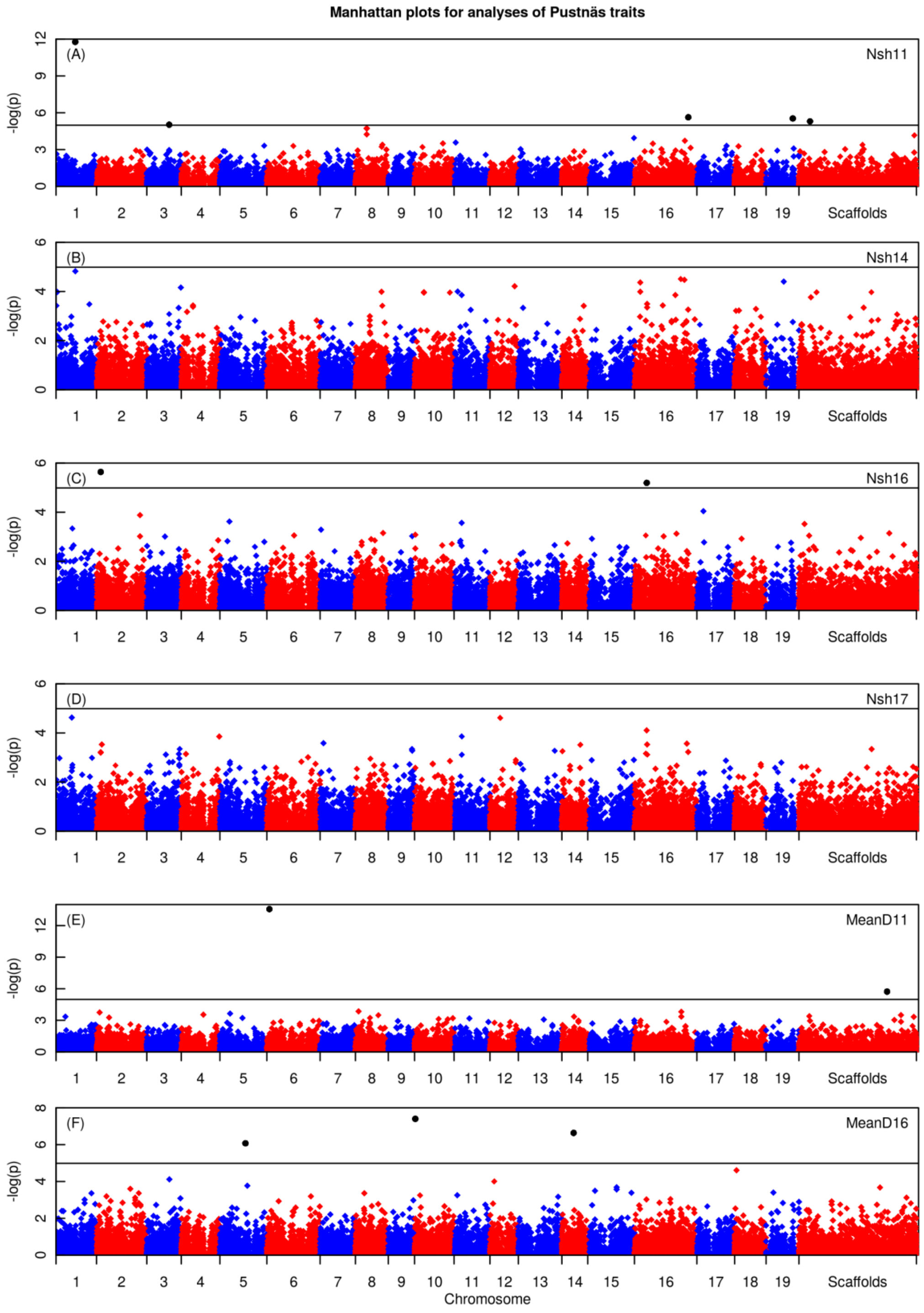

Figure S3. Manhattan plots showing the negative logarithms of *p*-values (*-log(p)*) of marker associations to the number of shoots (Nsh, subplots A-D) and mean shoot diameter (MeanD, subplots E-F) during the years 2011, 2014, 2016 and 2017 in Pustnäs. The chosen significance threshold (p=1.03·10^{-5}) is shown as a black line and associations included in the final selected model are highlighted as black circles.

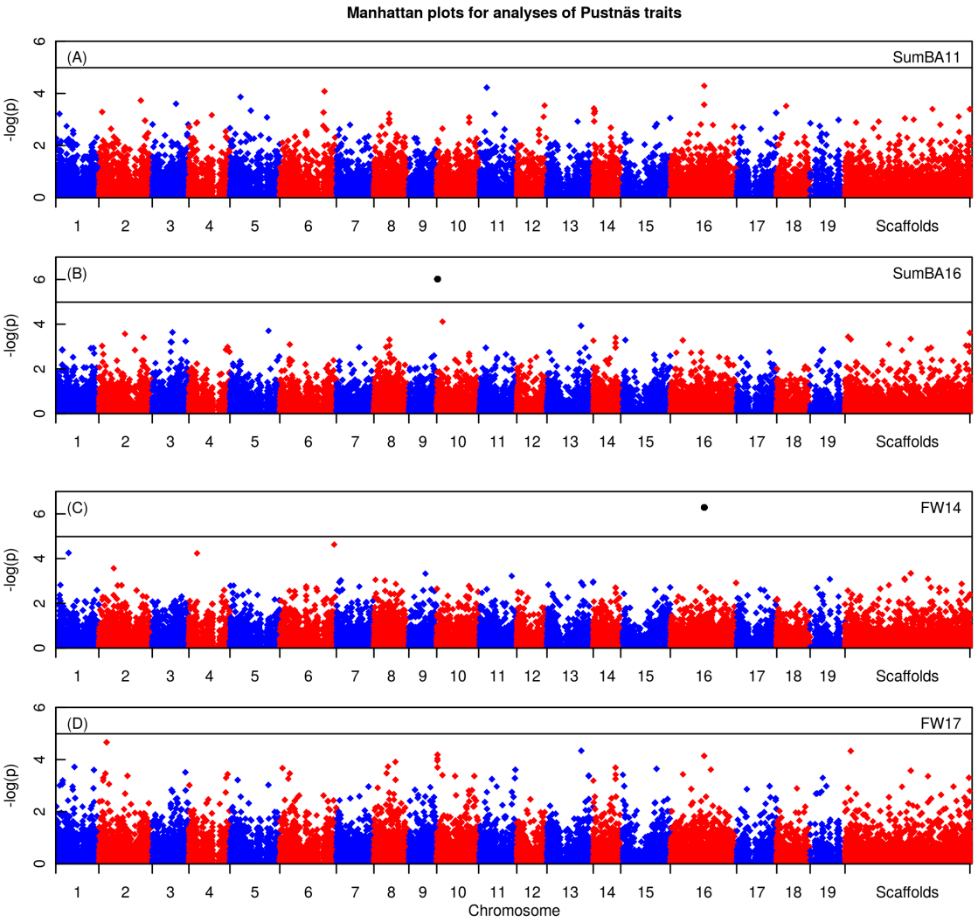

Figure S4. Manhattan plots showing the negative logarithms of *p*-values (*-log(p)*) of marker associations to the summed basal area (SumBA, subplots A-B) and harvest biomass fresh weight (FW, subplots C-D) during the years 2014 and 2017 in Pustnäs. The chosen significance threshold (p=1.03·10^{-5}) is shown as a black line and associations included in the final selected model are highlighted as black circles.

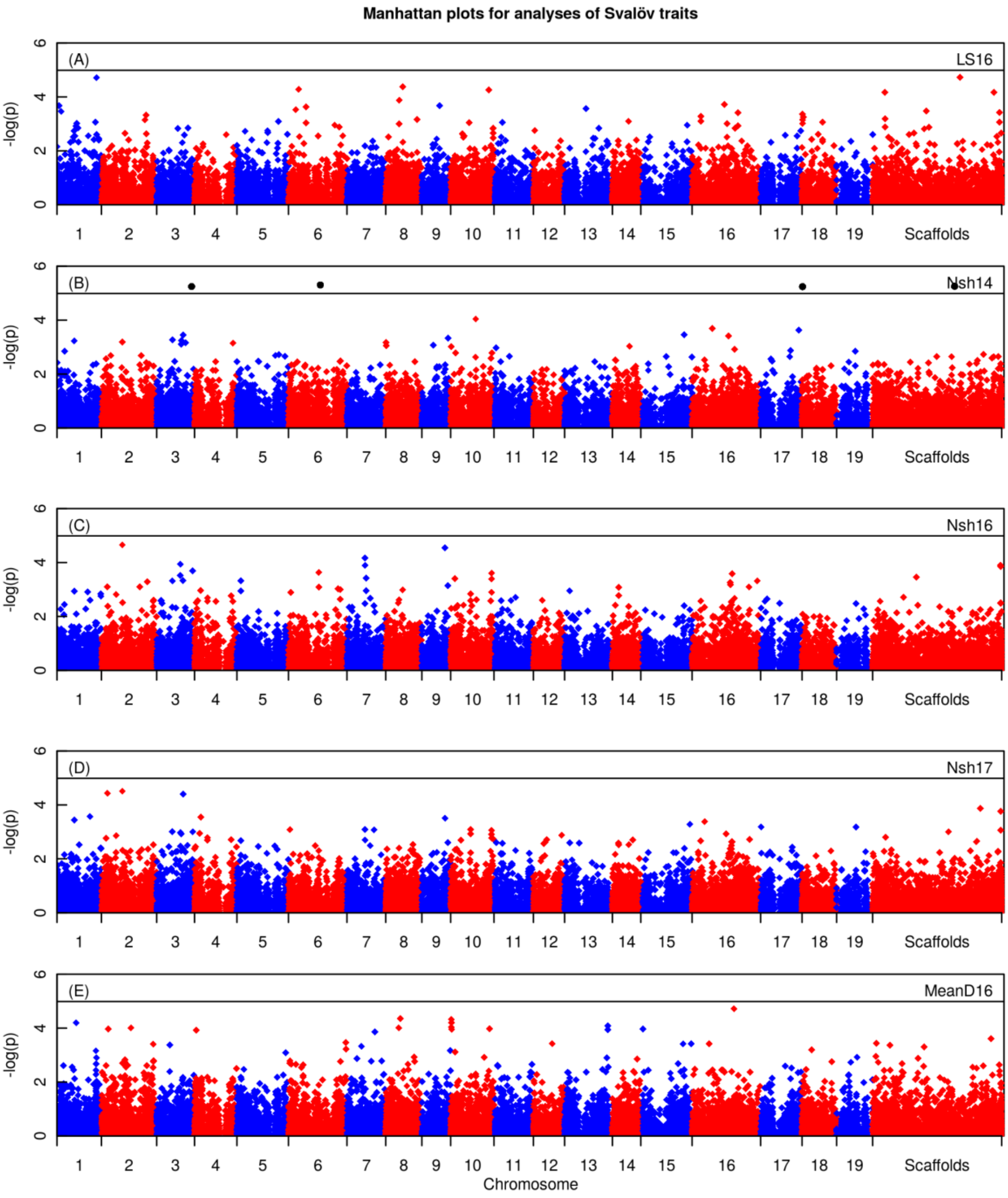

Figure S5. Manhattan plots showing the negative logarithms of *p*-values (*-log(p)*) of marker associations to the leaf senescence (LS, subplot A), number of shoots (Nsh, subplots B-D) and mean diameter (MeanD, subplot E) during the years 2014, 2016 and 2017 in Svalöv. The chosen significance threshold (p=1.03·10^{-5}) is shown as a black line and associations included in the final selected model are highlighted as black circles.

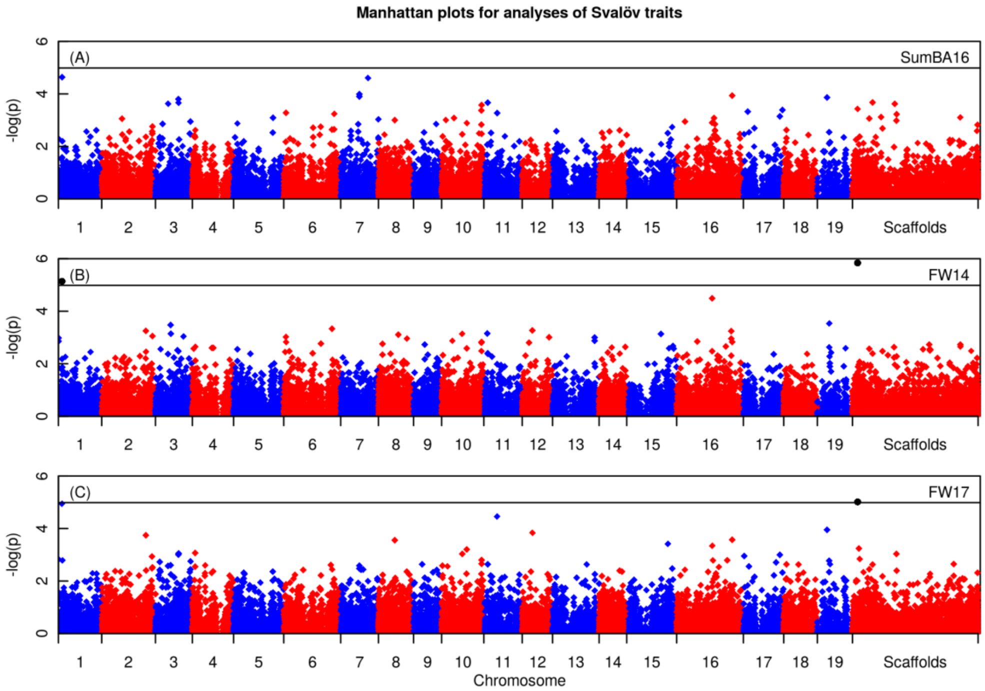

Figure S6. Manhattan plots showing the negative logarithms of *p*-values (*-log(p)*) of marker associations to the summed basal area (SumBA, subplot A) and harvest biomass fresh weight (FW, subplots B-C) during the years 2014, 2016 and 2017 in Svalöv. The chosen significance threshold (p=1.03·10^{-5}) is shown as a black line and associations included in the final selected model are highlighted as black circles.

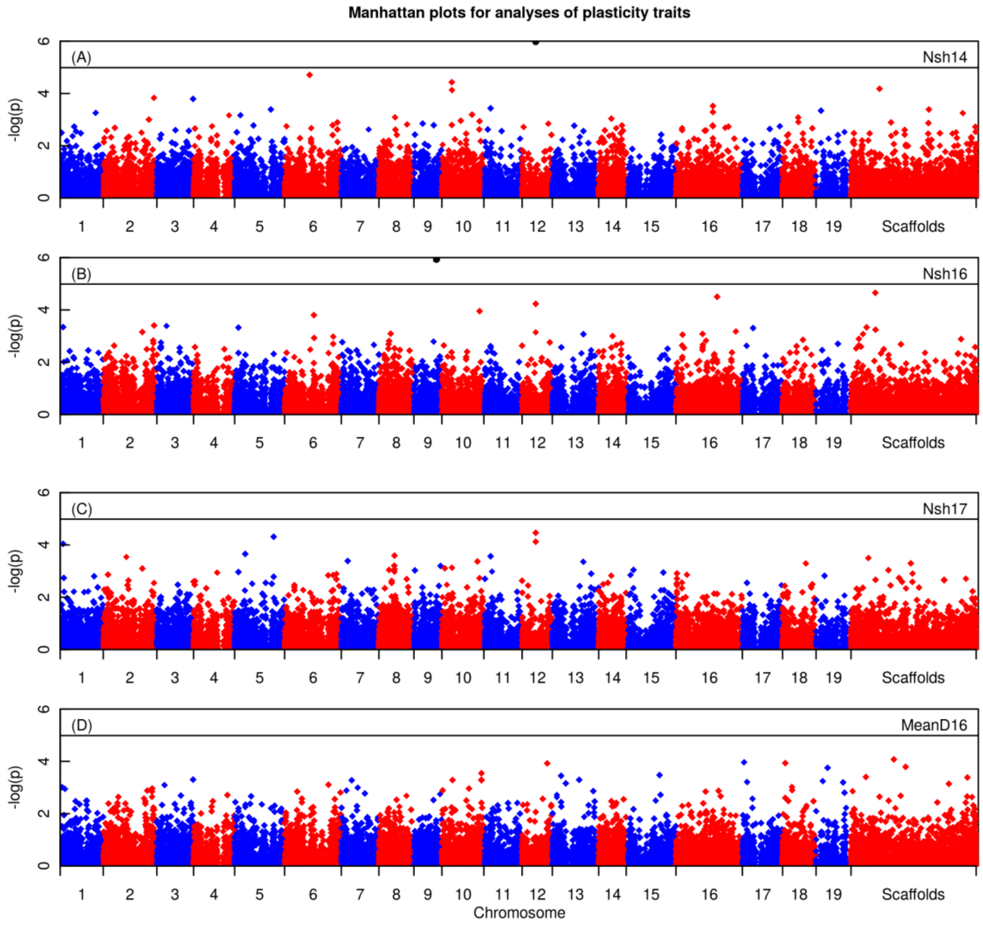

Figure S7. Manhattan plots showing the negative logarithms of *p*-values (*-log(p)*) of marker associations to the plasticity of number of shoots (Nsh, subplots A-C) and plasticity of mean shoot diameter (MeanD, subplot D) between Pustnäs and Svalöv during the years 2014, 2016 and 2017. The chosen significance threshold ($p = 1.03 \cdot 10^{-5}$) is shown as a black line and associations included in the final selected model are highlighted as black circles.

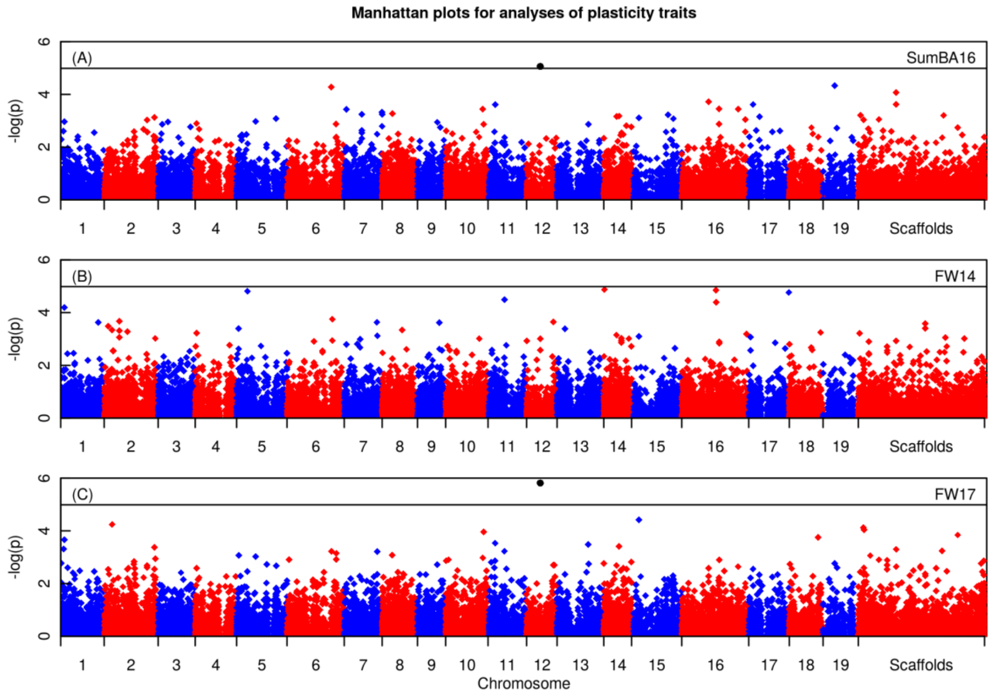

Figure S8. Manhattan plots showing the negative logarithms of *p*-values (*-log(p)*) of marker associations to the plasticity of summed basal area (SumBA, subplot A) and plasticity of harvest biomass fresh weight (FW, subplots B-C) between Pustnäs and Svalöv during the years 2014, 2016 and 2017. The chosen significance threshold (p=1.03·10^{-5}) is shown as a black line and associations included in the final selected model are highlighted as black circles.

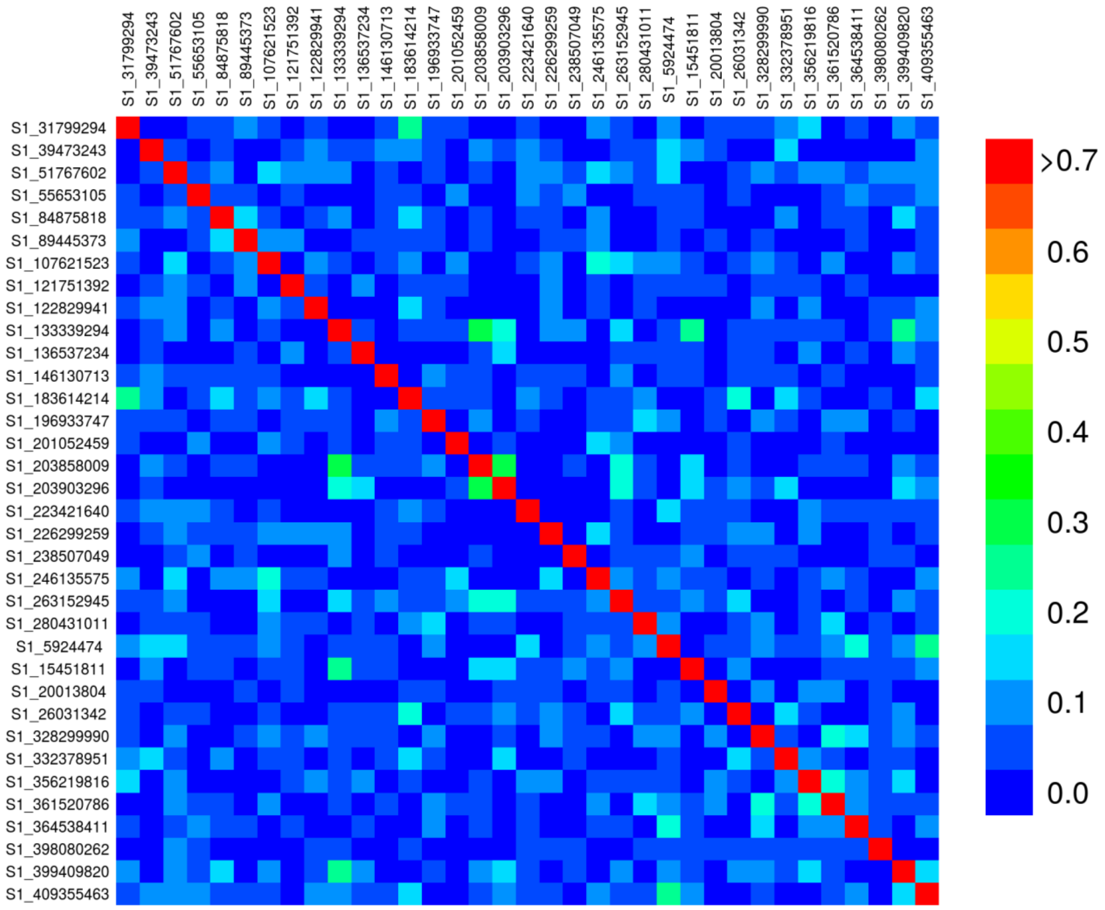

Figure S9. Linkage disequilibrium correlation matrix estimated between markers that were found to be significantly (p<0.2 after Bonferroni-correction) associated to traits.
